# A Different Microbiome Gene Repertoire in the Airways of Cystic Fibrosis Patients with Severe Lung Disease

**DOI:** 10.3390/ijms18081654

**Published:** 2017-07-29

**Authors:** Giovanni Bacci, Alessio Mengoni, Ersilia Fiscarelli, Nicola Segata, Giovanni Taccetti, Daniela Dolce, Patrizia Paganin, Patrizia Morelli, Vanessa Tuccio, Alessandra De Alessandri, Vincenzina Lucidi, Annamaria Bevivino

**Affiliations:** 1 Department of Biology, University of Florence, Florence 50019, Italy; giovanni.bacci@unifi.it (G.B.); alessio.mengoni@unifi.it (A.M.); 2 Cystic Fibrosis Microbiology and Cystic Fibrosis Center, “Bambino Gesù” Children’s Hospital and Research Institute, Rome 00165, Italy; ersilia.fiscarelli@opbg.net (E.F.); vanessa.tuccio@opbg.net (V.T.); vincenzina.lucidi@opbg.net (V.L.); 3 Centre for Integrative Biology, University of Trento, Trento 38123, Italy; nicola.segata@unitn.it; 4 Department of Pediatric Medicine, Cystic Fibrosis Center, Anna Meyer Children’s University Hospital, Florence 50139, Italy; giovanni.taccetti@meyer.it (G.T.); daniela.dolce@meyer.it (D.D.); 5 Territorial and Production Systems Sustainability Department, ENEA, Italian National Agency for New Technologies, Energy and Sustainable Economic Development, Casaccia Research Center, Rome 00123, Italy; patrypaganin@yahoo.it; 6 Cystic Fibrosis Center, IRCCS G. Gaslini Institute, Genoa 16146, Italy; patriziamorelli@gaslini.org (P.M.); aledeales@gmail.com (A.D.A.)

**Keywords:** cystic fibrosis, lung disease, lung microbiome, shotgun metagenomics, bioinformatics, metabolic pathways, virulence genes

## Abstract

In recent years, next-generation sequencing (NGS) was employed to decipher the structure and composition of the microbiota of the airways in cystic fibrosis (CF) patients. However, little is still known about the overall gene functions harbored by the resident microbial populations and which specific genes are associated with various stages of CF lung disease. In the present study, we aimed to identify the microbial gene repertoire of CF microbiota in twelve patients with severe and normal/mild lung disease by performing sputum shotgun metagenome sequencing. The abundance of metabolic pathways encoded by microbes inhabiting CF airways was reconstructed from the metagenome. We identified a set of metabolic pathways differently distributed in patients with different pulmonary function; namely, pathways related to bacterial chemotaxis and flagellar assembly, as well as genes encoding efflux-mediated antibiotic resistance mechanisms and virulence-related genes. The results indicated that the microbiome of CF patients with low pulmonary function is enriched in virulence-related genes and in genes encoding efflux-mediated antibiotic resistance mechanisms. Overall, the microbiome of severely affected adults with CF seems to encode different mechanisms for the facilitation of microbial colonization and persistence in the lung, consistent with the characteristics of multidrug-resistant microbial communities that are commonly observed in patients with severe lung disease.

## 1. Introduction

Subjects affected by cystic fibrosis (CF) experience a progressive loss of pulmonary functions accompanied by an increased burden of chronic infections. The respiratory microbial composition is particularly relevant for patients with CF. In fact, bacterial lung infections reduce life expectancy in patients with CF (the median predicted survival age is equal to 41.6, as reported in the Cystic Fibrosis Foundation Patient Registry [1]), and represent the primary cause of morbidity and mortality in CF patients [2]. In the last decade, the emergence of high-throughput sequencing approaches, coupled with the development of new bioinformatics pipelines designed to cope with metagenomics data, has revolutionized the study of complex bacterial communities such as the airway microbiota of CF patients, thereby improving our understanding of this largely unknown “microbial black-box”. This understanding of how a bacterial community changes over time and over the course of CF disease progression has revealed a different bacterial community structure upon pulmonary exacerbations (such as a reduced bacterial diversity and an increasingly conserved community composition) coupled with a decline in pulmonary health, antibiotic treatment, and patient age [3,4,5,6,7,8,9,10,11]. No overall changes in total bacterial density with exacerbation was observed [12], suggesting that shifts in the relative abundance of bacterial community members, rather than changes in total bacterial density, are more likely to be associated with alterations in clinical state [12].

In previous studies [13,14], we provided new insights into the features of the bacterial communities of patients with CF, improving our current knowledge about the airway microbiota composition and polymicrobial interactions in patients following a severe decline in lung function and with different lung disease statuses (normal/mild vs. moderate vs. severe). Although the description of taxonomic groups colonizing the airways of CF patients has helped in the understanding of how bacterial species change during disease progression, the overall gene content harbored by these communities remains largely unknown. Since metabolic and functional features of a microbial species are the dominant factors determining its ability to survive in a given environment (e.g., CF patients’ lungs) [15], untargeted metagenomic approaches may provide deep insights into the microbial CF lung metagenome, permitting the identification of gene sets involved in functional pathways associated with a worsening clinical condition [16]. 

In the present study, we performed a deep metagenomic investigation to probe the lung microbiome of twelve CF patients with mild (Forced expiratory volume in one second (FEV_1_) > 70%) and severe (FEV_1_ < 40%) lung disease. We aimed to investigate whether patients with different disease severities may indeed have a different representation of “keystone genes” in their lung microbiota. To the best of our knowledge, only a few works have already inspected the CF microbiome through shotgun metagenomes, and most of them are case reports on a limited number of patients [17,18] or on specific metabolic functions [19]. Moving away from taxonomic inventories towards a better understanding of CF microbiome genes opens a new avenue for the identification of the microbial gene repertoire associated with CF lung disease.

## 2. Results

### 2.1. Clinical Characteristics of Enrolled Patients and Culture-Based Diagnostic Microbiology

The clinical status of the individual subjects is reported in Table 1. The study cohort includes twelve patients (aged 18–46 years; median = 28) with CF who were in stable clinical conditions, without any pulmonary exacerbation and not undergoing antibiotic therapy (*i.v.* or oral) in the previous four weeks before specimen collection. The normal/mild (FEV_1_  > 70% predicted) and severe (FEV_1_  <  40% predicted) groups also differed in the extent of repeated antibiotic exposure and in their mean age being equal to (mean ± standard error) 25.33 ± 2.216 and 33.83 ± 3.146 years in the normal/mild and the severe group, respectively (two-tailed *t*-test; *p* value = 0.0313). In addition, the severe group tended to receive more uniform antimicrobial agents by the inhalation of aerosolised antibiotics during maintenance therapy, such as aerolized colistimethate and azithromycin, with respect to the normal/mild group (Table 1). Traditional culture-based diagnostic microbiology revealed the presence of *Pseudomonas aeruginosa* and *Staphylococcus aureus*, being the dominant bacterial species in patients with severe and mild respiratory function, respectively (Table S1). Other minor taxa were *Achromobacter xylosoxidans*, *Rothia mucilaginosa*, *Veillonella parvula*, *Stenotrophomonas maltophilia*, *Gemella sanguinis* and *Escherichia coli.*

### 2.2. Metabolic Community Structure between Patient Groups

**Figure 1:**
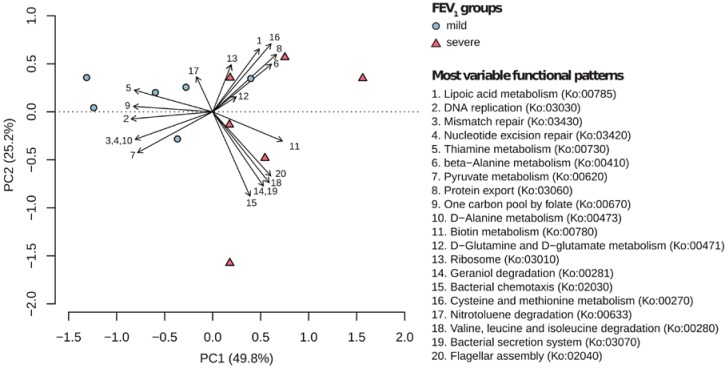
Principal component analysis (PCA) based on the top twenty metabolic patterns. Each number corresponds to a pathway reported in the figure legend, whereas each point corresponds to a different patient. Point shape reflects patient groups.

Sequencing yielded > 15 M paired-end sequences for each sample, with 1–5% of them being of putative microbial origin (the remaining being human DNA). This confirmed that human contamination still represents a considerable drawback in the metagenomic analysis of human sputum samples, as already reported in other metagenomics studies [20,21]. From 12 samples, a total of 5.4 M microbial sequences (mean ± standard error per sample, 453,824 ± 41,349) were included in the analysis (Table S2). The HMP Unified Metabolic Analysis Network (HUMAnN) analysis revealed 47 pathways which were differently distributed across all samples (Figure S1). The principal component analysis (PCA) analysis on metabolic and regulatory data (Figure 1) explained almost 80% of the total variance reporting a distinct sample distribution for normal/mild and severe patient groups. 

**Figure 2:**
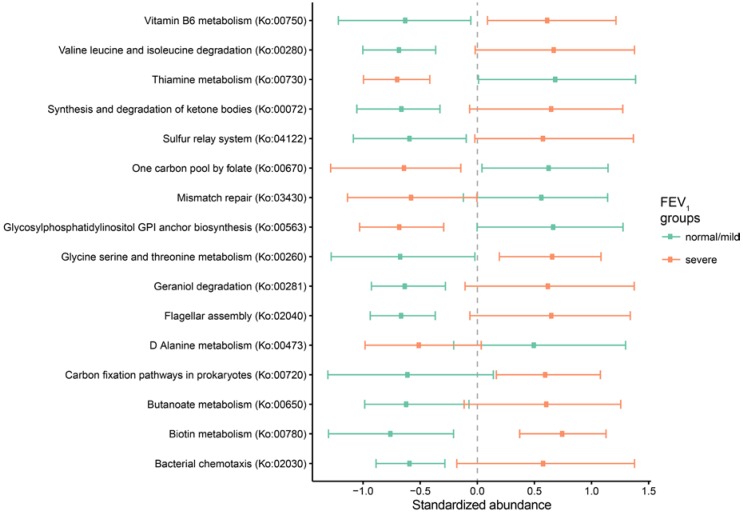
Differences in metabolic and regulatory pathways detected with HMP Unified Metabolic Analysis Network HUMAnN. Colors indicate statistically significant differences (*p*-value < 0.05) after Kruskal–Wallis one-way analysis of variance. Values indicate mean and one standard deviation (bars). Standardized abundances (x axis) were calculated as: [x − m(x)]/sd(x), where “SD” is the standard deviation and “m” is the mean value.

In particular, the separation of normal/mild samples from severe samples was largely driven by 6 pathways: biotin metabolism (Ko:00780), geraniol degradation (Ko:00281), bacterial chemotaxis (Ko:02030), valine leucine and isoleucine degradation (Ko:00280), the bacterial secretion system (Ko:03070), and flagellar assembly (Ko:02040). The Kruskal–Wallis one-way analysis of variance confirmed this result with five out of six previously identified pathways displaying higher values in the severe group patients compared to the normal/mild ones (Figure 2). Overall, 31 pathways were equally distributed between the two groups considered (Figures S2 and S3), whereas 16 pathways were differentially distributed in the two groups, being found mainly in patients with normal/mild (5 pathways) and severe disease (11 pathways), respectively (Figure 2).

### 2.3. Metagenomic Functional Differences Suggest Distinct Ecological Roles within the Cystic Fibrosis Microbiome

**Figure 3:**
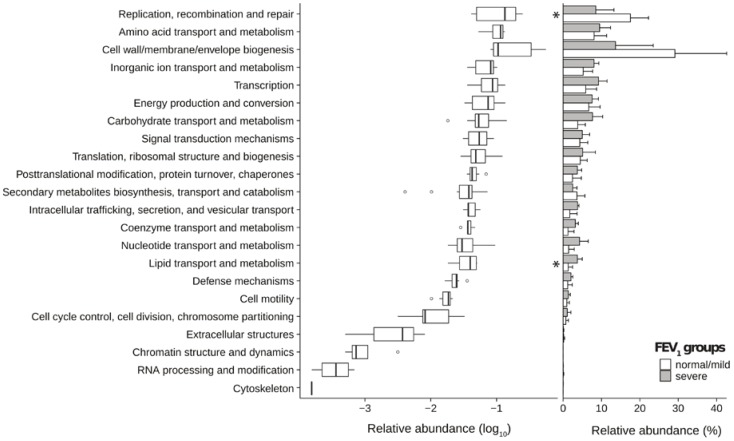
Distribution of functional categories obtained with an evolutionary genealogy of genes: non-supervised orthologous groups (eggnog) analysis. Box plots for each eggNOG category found for both groups of patients are reported in the left panel, whereas relative abundances for each group are reported in the right panel (bars represent the average value and error bars indicate the standard error for each measure). Significant differences between normal/mild and severe groups are flagged with an asterisk. Boxes denote the interquartile range (IQR) between the 25th and the 75th percentile (first and third quartiles), whereas the inner line represents the median. Whiskers represent the lowest and highest values within 1.5 times IQR from the first and third quartiles. Outliers are reported using white circles.

In order to establish a set of gene putatively available in CF lung communities, metagenomic reads were assembled into contigs. We were able to assemble an average of 1655 contigs (s.e. 526) per sample (N50 values ranging from 645 to 1655) (Figure S4 and Table S2). The gene-calling step predicted a mean of 5014 open reading frames (ORFs) with a standard error of 1266. The functional potential harbored by microbiomes was inferred, predicting gene functions by homology using the evolutionary genealogy of genes: non-supervised orthologous groups (eggNOG) database (Figure 3). 

**Figure 4:**
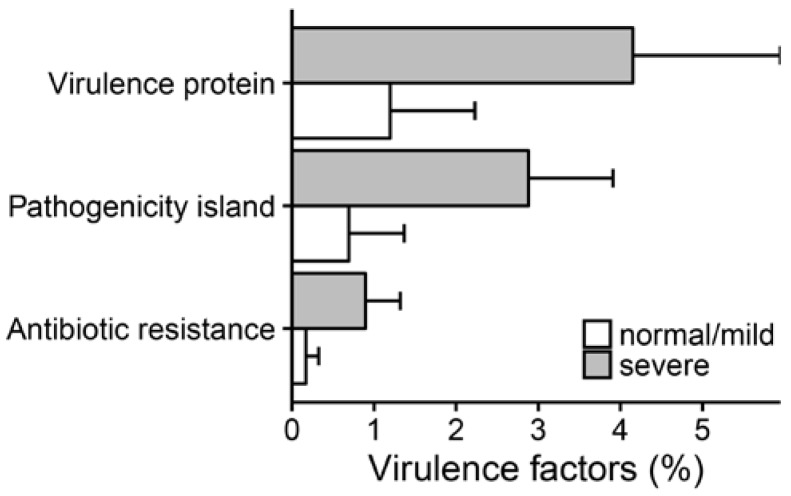
Virulence factor distribution across patient groups. Only factors displaying a significant (Student’s *t*-test *p*-value ≤ 0.05) diverging distribution are reported. The bars represent the average value for each category whereas the error bars indicate the standard error.

Both patient groups showed a massive presence of transporter proteins, with the severe group reporting higher values than the normal/mild group. The Transporter Classification Database (TCDB) analysis highlighted the possible presence of antibiotic resistance proteins indicating an imbalanced distribution of the multidrug resistance efflux pumps of the ATP-binding cassette (ABC) and resistance–nodulation–cell division (RND) super-families between the two groups of patients. In particular, patients with severe disease showed a high presence of both ABC and RND super-families members (Figure S5), thus suggesting the presence of efflux-mediated antibiotic resistance mechanisms. Virulence protein counts were normalized using the total number of ORFs predicted in each sample. Virulence (and virulence-related) factors displaying a significant difference between groups (two-tailed Student’s *t*-test *p*-value ≤ 0.05, alpha = 0.05) are shown in Figure 4 (individual values for each patient are reported in Figures S6 and S7). 

**Figure 5:**
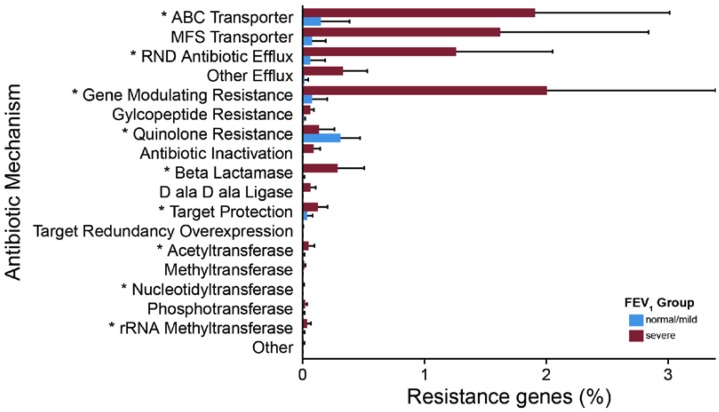
Antibiotic resistance gene differentiate patient groups. Bar charts report the percentage values of antibiotic resistance gene detected. Significant differences are reported with one asterisk (*p*-value < 0.05). *P* values were obtained through a Student’s *t*-test on the number of genes detected. Bars were drawn by computing the average percentage value of each resistance category whereas error bars are reported using standard errors.

A larger presence of virulence factors was found in patients with severe disease, with antibiotic resistance as the third most abundant category. The Resfams analysis [22] suggests that the microbes in CF airways encode a diverse set of antibiotic resistance mechanisms; among them, the multidrug resistance efflux pumps are the most represented (Figure 5). Overall, 17 out of 18 antibiotic resistance categories showed higher values in patients with severe disease, with 8 of them reporting significant differences. As expected, efflux mediated systems were the most abundant categories, all reporting higher values for severe patients than for normal/mild ones (Figure 5 and Figure S8). Our results are supported by culture-based analysis, which reveals the presence of multi-drug resistant bacteria in the severe group (Table S1).

### 2.4. Relationship between Functional Potential and Taxonomic Microbial Composition

To further characterize the airway microbiome of CF patients, the correlation between the taxonomic and functional structure of the microbial community was examined. The dissimilarity of the taxonomic community was significantly correlated with the functional community dissimilarity (Mantel test with Pearson correlation coefficient and 1000 permutations; *p*-values < 0.05), implying a relation between the species distribution patterns and the functional distribution of the whole airway community. Otherwise, the metabolic pathway distribution (Kyoto Encyclopedia of Genes and Genomes (KEGG) analysis) was poorly correlated with other features, reporting significant correlations only with antibiotic resistance gene distribution and taxonomic structure (Table S3). 

To further identify the driving forces for the taxonomic distribution, the relative influence of functional genes, metabolic pathway distribution, virulence factors, and antibiotic resistance genes were rendered by using boosted tree models on principal components derived from each feature. Principal components with an eigenvalue > 1 in total accounted for more than 85% of the total variance and were therefore chosen for training boosted regression trees (Table S4). In boosted tree models, measures of relative influence quantify the importance of a given predictor; the variables that have a weak effect on prediction therefore have, by extension, scarcely an influence on the variable that the model tries to predict [23]. Therefore, factors that have a minimal relative influence in predicting the abundance of a given taxon are poorly connected with the distribution of that taxon. Although the use of orthogonal composite variables (as reported in the method section) reduced type I errors, the contribution of each original variable cannot be inferred directly. To partially overcome this limitation, the most dominant variables contributing to each component (top-50% eigenvalues of K, E, A, and V) were reported in Table S4. 

**Figure 6:**
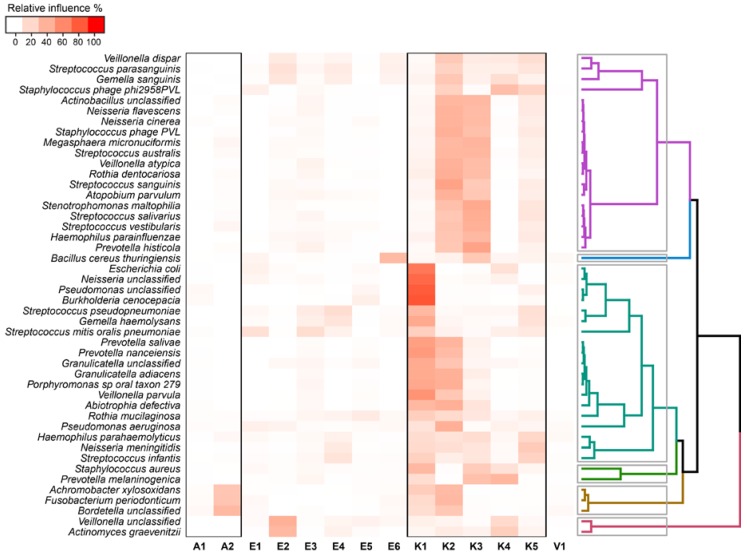
Relative influence of Kyoto Encyclopedia of Genes and Genomes (KEGG) pathways (K1 to K5), gene frequencies (evolutionary genealogy of genes: non-supervised orthologous groups (eggnog) database, E1 to E6), antibiotic resistance genes (Resfam database A1 and A2), and virulence factors (microbial database of protein toxins, virulence factors and antibiotic resistance genes for bio-defense applications (MvirDB), V1) for taxonomic structure of microbial lung community evaluated through “booster regression trees” models. The relative influence of different principal component to each bacterial species detected was clustered with the unweighted pair-group method with arithmetic mean (UPGMA) method based on Pearson’s correlation and a tree was reported on the right side of the plot. Boxes report the main group of taxa detected through a cluster analysis.

Based on the results reported in this study, the metabolic pathway distribution inferred with KEGG (K) was the major factor able to predict the microbial taxonomic structure, whereas the gene distribution obtained with eggNOG (E) had little influence on the whole community, affecting only the distribution of a limited number of species (Figure 6).

Surprisingly, antibiotic resistance genes (A) were related to three bacterial species only: *Achromobacter xylosoxidans*, *Fusobacterium periodonticum*, and an unclassified member of the *Bordetella* genus. *A. xylosoxidans* strains, intrinsically resistant to many classes of antimicrobial agents, have been associated with CF [24,25], as well as *F. periodonticum*, which has been reported to increase its presence during exacerbation [26]. Conversely, virulence factors (V) (mainly comprising genes previously identified in pathogenicity islands, virulence proteins, and antibiotic resistance genes Figure S7 and Figure 4) were not related to any particular species reporting the lowest values of relative influence.

## 3. Discussion

In an endeavor to better understand the complexity of CF microbiomes in patients with high/low pulmonary function, we performed a metagenomics investigation of the bacterial communities in the airways of patients with CF focusing on the identification of a set of functional features associated with different lung disease statuses in the present study.

Sputum specimens, which represent by far the most widely used sample to access the airway microbiota of CF disease [27,28,29,30], were collected from twelve patients with CF. As recently indicated by Dickson and colleagues [31], although use of sputum can introduce an additional risk of upper airway contamination, the presence of oropharyngeal microbiota does not obscure the meaningful microbial signal in sputum which is correlated with established indices of lung health. To date, in CF metagenomics, published studies have focused on the sputum derived from CF patients [17,32,33,34]. The shotgun metagenomics approach, which provides the gene catalogue of the microbial community, allows an insight into some functional features to be gained (e.g., the presence of metabolic pathways and potential antibiotic resistance genes), in addition to accurate species-level taxonomic assignments.

In the first metagenomic study performed by Willner and colleagues [33], the airways of diseased and non-diseased individuals showed a potentially different microbial community metabolism (inferred from the recovered gene catalogue of the microbiome), suggesting that the community metabolism is dynamic and variable among patients differing in their health status. In the present work, the abundances of metabolic pathways encoded by microbes inhabiting CF airways of patients with high/low pulmonary function were reconstructed from the metagenome. The metabolic pathway distribution described in this work was in accordance with the one described in other studies [19,32], with pathways related to amino acid catabolism, nucleotide metabolism, and stress responses reporting high values in both normal/mild and severe patients. Several pathways were uniformly distributed among patients, regardless of their clinical condition, reflecting a large set of core functions typical of host-associated microbes. Indeed, according to the previous studies mentioned above [19,32], there was little variation in the metagenome pathway distribution, with sixteen pathways only reporting different abundance values between the two groups of patients inspected here. Interestingly, we identified a set of metabolic pathways correlated with the worsening of patients’ clinical conditions; in particular, two pathways, the *bacterial chemotaxis pathway* (Ko:02030) and the *flagellar assembly pathway* (Ko:02040), were reported as clinically relevant according to previous works [35,36,37,38,39]. Indeed, one pathway was associated with *Pseudomonas aeruginosa* lung infection (bacterial chemotaxis pathway), whereas the second (flagellar assembly pathway) was classified as a potent mediator of virulence in Gram-negative bacteria such as *P. aeruginosa* strains. Moreover, the *motB* gene (a component of the flagellar assembly pathway) is known to aid bacterial chemotaxis and flagellar assembly; also, its product (a membrane protein, flagellar motor protein) was recognized as a potential novel vaccine target in *Vibrio cholerae* [40]. We can hypothesize that the difference in the representation of the above-mentioned pathways (Ko:02030 and Ko:02040) could rely on both *P. aeruginosa* strain differences (between the two disease groups) as well as on the presence of other bacteria in the CF microbiome which carry the same gene sets. The presence of *P. aeruginosa* and other Gram-negative strains that could be responsible for the appearance of these pathways were also revealed by cultivation methods analysis, as reported in Figure S9. However, the limited number of patients analyzed in our study does not allow for definitive, statistically-based conclusion on this issue.

In addition to microbial genes involved in metabolic metabolism, genes involved in antibiotic resistance are also powerful indicators of the microbial community’s adaptation to the CF lung [17]. Indeed, the top-50% KEGG pathways (reported in Table S4) were mainly involved in the metabolism of amino acids and nucleotides and may be linked to bacterial proliferation and adaptation in the lungs. An increased presence of genes involved in both nucleotide and amino acid metabolism may, in fact, indicate a huge presence of both types of molecules which, in turn, may come from neutrophil extracellular traps, bacterial biofilms and the action of human and bacterial proteases [32,41,42,43,44]. Moreover, a large number of genes classified with eggNOG belonged to the transporter families. Their further analysis by using the Transporter Classification Database (TCDB) permitted us to identify a massive presence of families connected with antibiotic resistance mechanisms, especially in patients with severe lung disease. The presence of genes related to antibiotic resistance mechanisms was confirmed by the Hidden Markov model (HMM) (Resfams), which accurately predicts new resistance functions from sequence alone [22]. The efflux-mediated system, which represents the widespread drug resistance mechanism common to many microorganisms, was the most represented gene function. Interestingly, the presence of *P. aeruginosa* and *Staphylococcus aureus* was not related to the differential abundance of antibiotic resistance genes and virulence factors. Indeed, virulence and resistance genes have been found to be spread throughout the whole CF bacterial community and are poorly related to the presence of single taxa, suggesting that the emergence of these mechanisms should be attributed to a particular clinical condition (normal/mild or severe lung disease) of CF patients. In fact, significant differences in the percentage of total Antibiotic Resistance (AR) functions encoded in microbial genomes between normal/mild and severe groups were found. Overall, there was a significant AR mechanism enrichment in patients with severe lung disease due to several years of exposure to antimicrobial drugs. It is well known that several antimicrobial agents and complex regimens are used for prophylaxis, eradication, treatment of exacerbations, and chronic suppressive therapy [45,46], paving the way for the emergence and spreading of AR mechanisms throughout the lung microbial community. In particular, oral, intravenous, and inhaled antibiotic courses are often frequent and prolonged, especially with increasing age and declining pulmonary status [47] as those characterizing the patients with severe lung disease. As underlined by Zhao and colleagues [28], an antibiotic administered closer to the time of sampling would be expected to have a greater impact on the microbial community than that same antibiotic would have when administered at a longer interval from the sampling time. Even if, on the date of sampling and in the 30 days before sample collection, antibiotic administration through an IV route was not given to our patients, we can hypothesize that patients with severe lung disease, having an average higher age than those from the normal/mild group, experienced several years of exposure to antimicrobial drugs, leading to periodic selection for an AR microbiota and resulting in a higher frequency and diversity of AR genes in their lung microbiota. The depth and diversity of AR genes uncovered by metagenomic studies in CF patients with different disease statuses brings to light the need for new strategies to combat antibiotic-resistant pathogens [48]. As reported by King and colleagues [49], the metagenomic profiles of human microbiota are becoming increasingly characterized, and growing data suggests that imbalances of the microbiota could lead to a disease status [50].

## 4. Materials and Methods 

### 4.1. Ethics Statement

The study was approved by the Ethics Committees of Children’s Hospital and Research Institute Bambino Gesù (Rome, Italy), Cystic Fibrosis Center, Anna Meyer Children’s University Hospital (Florence, Italy) and G. Gaslini Institute (University of Genoa, Genoa, Italy) (Prot. N. 681 CM of 2 November 2012; Prot. N. 85 of 27 February 2014; Prot. N. FCC 2012 Partner 4-IGG of 18 September 2012). Informed written consent was given by all adult subjects before enrollment in the study. All sputum specimens were produced voluntarily. All procedures were performed in agreement with the “Guidelines of the European Convention on Human Rights and Biomedicine for Research in Children” and the Ethics Committee of the three CF Centers involved. All measures were obtained and processed ensuring patient data protection and confidentiality. 

### 4.2. Patients

Twelve patients with CF (aged 18–46 years), older than six years, were enrolled in the study between September 2012 and April 2013. The cohort consisted of clinically stable patients without any pulmonary exacerbation and who were not undergoing antibiotic therapy (*i.v.* or oral) in the previous four weeks before specimen collection [51,52]. Patients were treated according to the current standards of care [53] with at least four microbiological controls per year [54]. At each visit, clinical data collection and microbiological status (colonizing pathogens with available cultivation protocols) were performed according to the European CF Society standards of care [55]. Forced expiratory volume in one second as a percentage of predicted (%FEV_1_) is a key outcome of the monitoring of lung function in CF [56]. All enrolled patients had an absence of an acute pulmonary exacerbation. Patients were classified into two groups, “normal/mild” (FEV_1_ > 70%) and “severe” (FEV_1_ < 40%), by estimating their average annual FEV_1_ value on the basis of multiple spirometric measurements over the two years before their enrollment. FEV_1_ values were measured according to the American Thoracic Society (ATS) and the European Respiratory Society (ERS) standards [57,58]. The lower limit of age for subjects with “normal/mild” disease was 15 years old, while that of subjects with severe disease was 25 years old. The study was approved by the Ethics Committees of Children’s Hospital and Research Institute Bambino Gesù (Rome, Italy), Cystic Fibrosis Center, Anna Meyer Children’s University Hospital (Florence, Italy) and G. Gaslini Institute (University of Genoa, Genoa, Italy), as stated in the ethical statement. The demographic and clinical characteristics of patients are reported in Table 1. The number of exacerbations (as defined by a cluster of symptoms and signs as previously indicated [51,52]) was determined in the five years before the enrollment. 

### 4.3. Sample Processing

The microbiome analysis was performed on sputum samples. Upon expectoration, CF sputum samples were immediately treated for 15 min with Sputolysin (Calbiochem, La Jolla, CA, USA) in accordance with the manufacturer’s instructions and split into aliquots for culture and molecular analyses. Aliquots for culturable analysis were immediately examined, and the remaining aliquots were frozen and stored at −80 °C for subsequent DNA extraction and metagenomic analysis. Bacterial detection and identification were performed as previously reported [13]. The microbiological status of the individual subjects is reported in Table S2.

### 4.4. DNA Extraction Procedures and Sequencing

About 400 µl aliquots of frozen sputum were subjected to genomic DNA extraction using acetyl trimethylammonium bromide (CTAB) protocol, according to the procedure previously reported [59]. Sample aliquots were spun at 10,000× *g* to pellet cellular material. After the removal of the supernatant, cell pellets were re-suspended in 567 μL of autoclaved and 0.2 filtered TE pH 8 and incubated for 1 h at 37 °C with 30 μL 10% sodium dodecyl sulfate (SDS) and 3 μL 20 mg/mL Proteinase K (Sigma-Aldrich, St. Louis, MO, USA). Samples were then incubated for 10 min with 100 μL of 5 M NaCl prepared with sterile water and 80 μL of CTAB/NaCl solution (4.1 g NaCl, 10 g CTAB in 100 mL sterile water). Following incubation, extracts were purified using phenol chloroform extraction, and DNA was recovered by isopropanol precipitation. Pelleted DNA was washed twice with cold 70% ethanol, allowed to air dry, and re-suspended in 30 μL of sterile water. Quantity and integrity of DNA extracted were assessed by Qubit 2.0 fluorometer (Invitrogen, Life technologies) and gel electrophoresis, respectively. Library preparation and DNA sequencing were performed following the standard pipelines for Illumina HiSeq 2000, PE100 sequencing (Beijing Genomics Institute, BGI, Shenzhen, Guangdong, China), as described in Supplementary Information S1. Raw sequence data reported in this study have been deposited in the National Center for Biotechnology Information (NCBI) “Sequence Read Archive” (SRA) under the project accession PRJNA316056. 

### 4.5. Bioinformatic Analyses

Sequence quality was ensured by trimming reads using StreamingTrim 1.0 [60], with a quality cutoff of 20. Bowtie2 [61] was used to screen out human-derived sequences from metagenomic data with the latest version of the human genome available in the NCBI database (GRCh38) as reference. Sequences displaying a concordant alignment (a mate pair that aligns with the expected relative mate orientation and with the expected range of distances between mates) against the human genomes were then removed from all subsequent analyses. Metabolic and regulatory patterns were estimated using HUMAnN [62] and considered only those pathways with a coverage value ≥ 80%, whereas the taxonomic microbial community composition was assessed using MetaPhlAn2 [63]. Reads were assembled into contigs using the SPAdes microbial assembler [64] with automatic k-mer length selection. To establish an airway microbiome gene catalog [65], we first removed contigs smaller than 500bp and then used MetaGeneMark [66] to predict open reading frames (ORFs). Translated protein sequences obtained from assembled contigs were classified using Hmmer [67] with the eggNOG [68] database trained on bacterial sequences (bactNOG). Each protein was classified according to its best hit with an e-value lower than 0.001 as suggested in [69]. Proteins classified as transporters were further inspected using Basic Local Alignment Search Tool (BLAST) against the Transporter Classification Database (TCDB) [70] to obtain a more detailed classification. Similarly, the MvirDB [71] database was used to inspect the distribution of virulence factors among our samples. We classified each sequence based on its BLAST best hit with an e-value lower than 1 × 10^−20^ in order to minimize the number of alignments that could be found by chance. Proteins that did not match any reference sequence were excluded from the analysis. Finally, predicted proteins were screened for antibiotic resistance activity based on the workflow described in [22] and validated for the Resfams database. 

### 4.6. Statistical Analyses

All statistical analyses were implemented in R (R Core Team (2015), version 3.2.3, R Foundation for Statistical Computing, Vienna, Austria) with the help of various packages. The distribution of metabolic pathways was assessed using the principal component analysis (PCA) on normalized data using the “rda” function of the vegan package version 2.3.2 [72]. The 20 metabolic pathways that varied greatly between normal/mild and severe groups were selected through the similarity of the percentages analysis (“simper” function) and used for PCA analysis (vegan 2.3.2). The Kruskal–Wallis one-way analysis of variance was used to test whether the metabolic and regulatory pathways of different patient groups originated from distinct distributions using the “Kruskal” function of the agricolae package (version 1.2.3). The distribution of ortholog genes, virulence factors, and antibiotic resistance mechanisms was normalized by the total number of open reading frames (ORFs) detected for each sample, and the differences between normal/mild and severe groups were assessed using the two-tailed Student’s *t*-test for each category (“t.test” function). The correlation between two dissimilarity matrices, obtained from metagenomics data such as the metabolic pathway or gene distribution, was assessed by using the Mantel test (vegan 2.3.2) as reported in other studies [73,74]. Basically, each table was transformed into a dissimilarity matrix using the “Bray–Curtis” dissimilarity index. By doing so, all dissimilarity matrices were reduced to the same rank, making it possible to use the Mantel test to inspect the correlation between them. The influence of gene patterns, metabolic modules distribution, virulence factors, and antibiotic-resistance genes on the taxonomic microbial composition was evaluated using “boosted regression tree” models [23] with 5000 trees, 10-fold cross-validation, and three-way interactions (gbm package version 2.1.1). Testing multiple individual variables assumed to be correlated with each other may inflate type I errors due to the high number of comparisons made [75]. To reduce the number of comparisons, and therefore to minimize the number of false positive correlations [75], orthogonal composite variables were derived from principal component analysis (PCA) for each factor explored, retaining only those components with an eigenvalue > 1. The derived variables were used for training boosted regression trees, minimizing the number of comparisons and, thus, reducing potential type I errors. A hierarchical cluster analysis was performed based on the average linkage method with “Pearson’s distance” metrics (“hclust” function). All graphical representations of data were performed using the ggplot2 package version 2.0.0 [76]. To minimize the type I errors in multiple comparisons, p-values where adjusted using a false discovery rate (FDR).

## 5. Conclusions

Our results highlight that different pulmonary conditions in patients with CF co-occur with a different microbiome gene repertoire. In particular, an imbalanced distribution of virulence factors along with AR genes and metabolic pathways has been found. Understanding the role of the CF airway microbiome and detecting microbiome genes associated with lower lung function are key challenges for the delivery of new potential biomarkers for the management of bacterial infection in CF patients and the improvement of health care treatment. Our study was limited to twelve subjects; therefore, a larger scale study is needed to more completely characterize the spectrum of microbiome changes associated with the decreasing of pulmonary function. Longitudinal metagenomic analysis, which we plan to evaluate in ongoing studies, may help in understanding imbalances down to the single gene level, possibly helping to find new therapeutic strategies for targeting personalized disease phenotypes.

## Figures and Tables

**Table 1:** Demographic and clinical characteristics of patients with normal/mild (Forced expiratory volume in one second (FEV_1_) > 70%) and severe (FEV_1_ < 40%) lung disease status enrolled in the study.

Study ID	Age	Gender	CFTR Genotype	BMI	Average Annual FEV_1_% Value	Lung Disease Status	Number of Exacerbations in the Last 5 Years	Maintenance Antimicrobial Therapy ^1^
BS29	25	F	F508del/L1077P	23.1	72	normal/mild	20 (2–7)	AT
BS47	33	M	F508del/N1303K	23.8	94	normal/mild	5 (1–2)	AA, AC
MS1	30	F	G1244 E/G1244 E	22.9	83	normal/mild	5 (0–4)	AC
GNR19	18	M	F508del/F508del	21.4	80	normal/mild	9 (1–3)	None
GNR5	24	M	F508del/12491G>A	22.7	72	normal/mild	9 (0–3)	None
BNR22	22	F	F508del/G85E	22.1	81	normal/mild	23 (2–7)	AT, AZ
BS19	36	M	F508del/W1282X	24.9	37	severe	18 (3–4)	AC, AZ
BS51	36	M	F508del/2789+5G>A	21	38	severe	16 (2–6)	AC, AZ
BS85	34	M	F508del/1259insA	18.8	21	severe	15 (2–5)	AC, AZ
BNR15	46	F	F508del/F508del	19.7	37	severe	16 (2–4)	AC, AZ
BNR20	25	M	F508del/F508del	23.3	36	severe	36 (4–11)	AA, AC
BNR49	26	M	N1303K/G85E	19.9	29	severe	10 (1–3)	AC

^1^ AT, aerosolized tobramycin; AA, aerosolized aztreonam; AC, aerosolized colistimethate; AA, aerosolized aztreonam; AZ, azithromycin.

## Supplementary Materials

Supplementary materials can be found at www.mdpi.com/1422-0067/18/8/1654/s1.
