# Supplementary material for: A Different Microbiome Gene Repertoire in the Airways of Cystic Fibrosis Patients with Severe Lung Disease
Source: Int J Mol Sci. 2017 Jul 29;18(8):1654. doi: 10.3390/ijms18081654 (PMC5578044; doi:10.3390/ijms18081654)
Supplement: Supplementary file 1 [file ijms-18-01654-s001.pdf]

Supplementary Materials: A Different Microbiome Gene Repertoire in the Airways of Cystic Fibrosis Patients with Severe Lung Disease

S1. Library preparation and NGS sequencing

Qualified DNA samples were used to prepare sequence library with an insert size of 180 bp. First of all, purified DNA samples were sheared into smaller fragments by Nebulization technique. Then the overhangs resulting from fragmentation were converted into blunt ends by using T4 DNA polymerase, Klenow Fragment and T4 Polynucleotide Kinase. After adding an 'A' base to the 3' end of the blunt phosphorylated DNA fragments, adapters were ligated to the ends of the DNA fragments. After that, too short fragments were removed using Ampure beads. The qualified library was used for sequencing with 2G raw data output per sample and a length of 100bp per read. After raw data generation, low quality reads were removed. Data filtration was done by BGI custom scripts, and listed as follows:

1. removing reads with 3 N;
2. removing reads contaminated by adapter;
3. removing reads with a certain proportion of low quality bases (40% as default, parameter setting at 36 bp);
4. removing duplication contamination.

Finally, the clean data obtained were used for subsequent bioinformatic analysis. The library preparation procedures, as well as the sequencing pipeline described here were implemented and performed by the Beijing Genomics Institute (BGI, Shenzhen, Guangdong, China). Additional details about the bioinformatics pipeline used in this work including information about raw data control were reported in the main text (Material and Methods section) and in S2 Table.

S2. File to be uploaded to iPath site (http://pathways.embl.de/)

Supplementary Figure Legends and Tables

Figure S1. Metabolic and regulatory pathway distribution as obtained from HUMAnN analysis. Only pathways reporting a coverage values higher than 80% were reported.

Figure S2. Metabolic map drawn with iPath. Equally distributed pathways were reported in green, whereas pathways with a higher abundance in normal/mild and severe groups were reported in blue and red. In particular, pathways reporting higher values in patients belonging to the normal/mild group were reported in blue and pathways more represented in patients belonging to the severe group were reported in red. An interactive version of this plot can be generated using Supporting Information S2 directly from iPath site (http://pathways.embl.de/).

Figure S3. Regulatory distribution derived from the metabolic map as reported in S2 Fig. Different colors refer to different distributions as reported in S2 Fig legend. An interactive version of this plot can be generated using S2 Supporting Information directly from iPath site (http://pathways.embl.de/).

Figure S4. Contig length distribution. Boxplots were drawn reporting the interquartile range (IQR) between the 25th and the 75th percentile (first and third quartiles), whereas the inner line represents the median. Whiskers represent the lowest and highest values within 1.5 times IQR from the first and third quartiles Outliers were not reported.

Figure S5. Distribution patterns of transporter proteins as reported by TCDB. On the left part of the panel, the top 30 transporter categories were enlarged for easy reading. "m" and "s" correspond to "normal/mild" and "severe" groups.

Figure S6. Virulence factor distribution for each patient. Only factors displaying a significant (Student's t-test p-value ≤ 0.05) diverging distribution in the two groups of patients were reported.

Figure S7. Distribution patterns of virulence factors classified with MvirDB. The top 20 sub-categories were reported for each main-category.

Figure S8. Distribution of antibiotic resistance genes for each category detected at least in one sample. The percentage of resistance genes found has been reported for each patient.

Figure S9. Top-20 taxa detected with both NGS and culture

Table S1. Culture-based diagnostic microbiology of sputum from patients with CF.

Table S2. Number of metagenomic sequences obtained and processed for each patient.

Table S3. Mantel test P values and R statistics obtained comparing all biotic features. In the right part of the table, the values of the R statistics were reported whereas the P values were reported in the left part. T=taxonomic; K=KEGG pathways; E=gene frequencies (eggNOG database); V=virulence factors (MvirDB database); A=antibiotic resistance genes (Resfam database).

Table S4. Variable loadings on the PCs across samples. The top-50% variables were reported in bold.

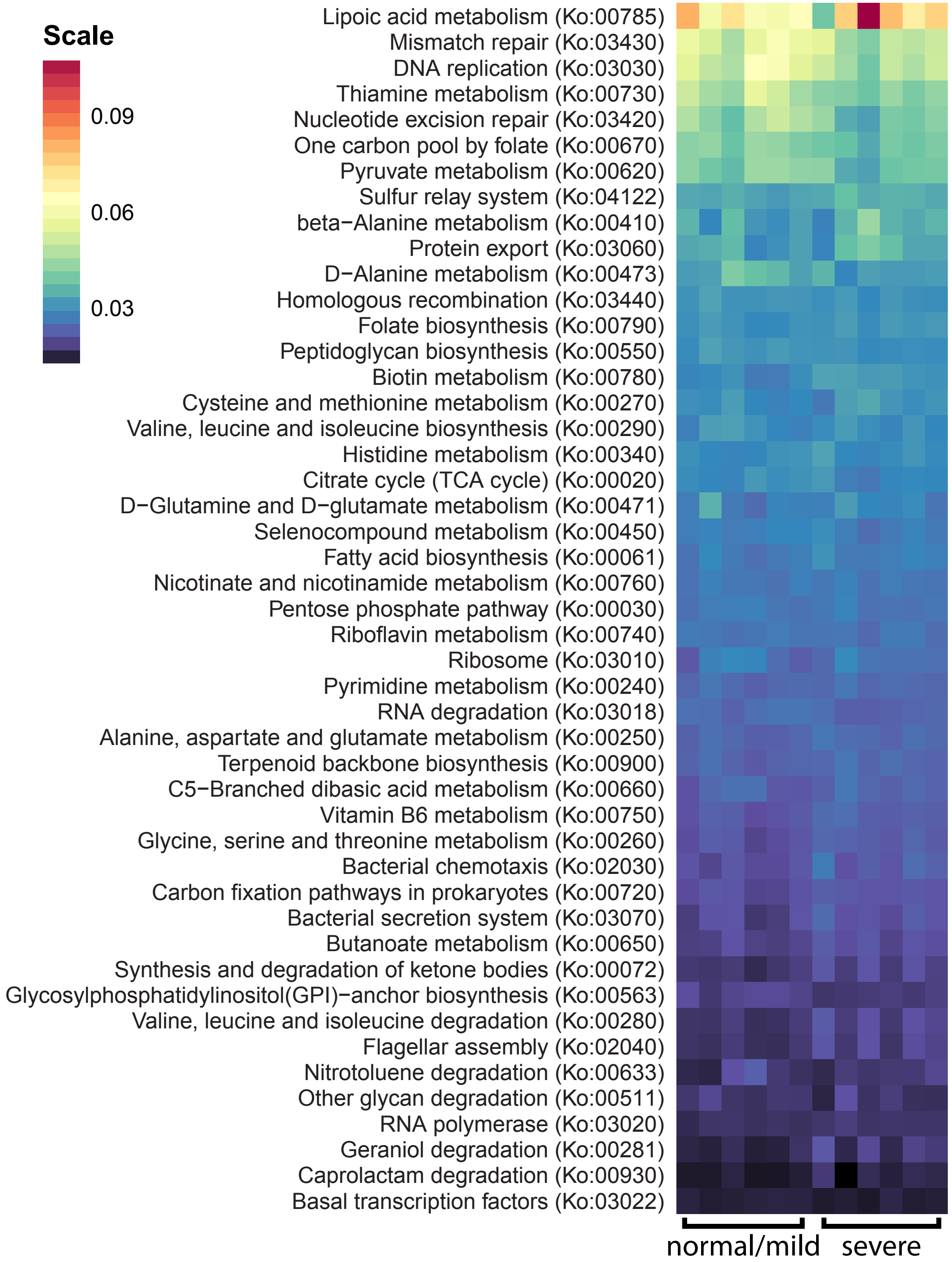

Scale
0.09
0.06
0.03
Lipoic acid metabolism (Ko:00785)
Mismatch repair (Ko:03430)
DNA replication (Ko:03030)
Thiamine metabolism (Ko:00730)
Nucleotide excision repair (Ko:03420)
One carbon pool by folate (Ko:00670)
Pyruvate metabolism (Ko:00620)
Sulfur relay system (Ko:04122)
beta−Alanine metabolism (Ko:00410)
Protein export (Ko:03060)
D−Alanine metabolism (Ko:00473)
Homologous recombination (Ko:03440)
Folate biosynthesis (Ko:00790)
Peptidoglycan biosynthesis (Ko:00550)
Biotin metabolism (Ko:00780)
Cysteine and methionine metabolism (Ko:00270)
Valine, leucine and isoleucine biosynthesis (Ko:00290)
Histidine metabolism (Ko:00340)
Citrate cycle (TCA cycle) (Ko:00020)
D−Glutamine and D−glutamate metabolism (Ko:00471)
Selenocompound metabolism (Ko:00450)
Fatty acid biosynthesis (Ko:00061)
Nicotinate and nicotinamide metabolism (Ko:00760)
Pentose phosphate pathway (Ko:00030)
Riboflavin metabolism (Ko:00740)
Ribosome (Ko:03010)
Pyrimidine metabolism (Ko:00240)
RNA degradation (Ko:03018)
Alanine, aspartate and glutamate metabolism (Ko:00250)
Terpenoid backbone biosynthesis (Ko:00900)
C5−Branched dibasic acid metabolism (Ko:00660)
Vitamin B6 metabolism (Ko:00750)
Glycine, serine and threonine metabolism (Ko:00260)
Bacterial chemotaxis (Ko:02030)
Carbon fixation pathways in prokaryotes (Ko:00720)
Bacterial secretion system (Ko:03070)
Butanoate metabolism (Ko:00650)
Synthesis and degradation of ketone bodies (Ko:00072)
Glycosylphosphatidylinositol(GPI)−anchor biosynthesis (Ko:00563)
Valine, leucine and isoleucine degradation (Ko:00280)
Flagellar assembly (Ko:02040)
Nitrotoluene degradation (Ko:00633)
Other glycan degradation (Ko:00511)
RNA polymerase (Ko:03020)
Geraniol degradation (Ko:00281)
Caprolactam degradation (Ko:00930)
Basal transcription factors (Ko:03022)
normal/mild
severe

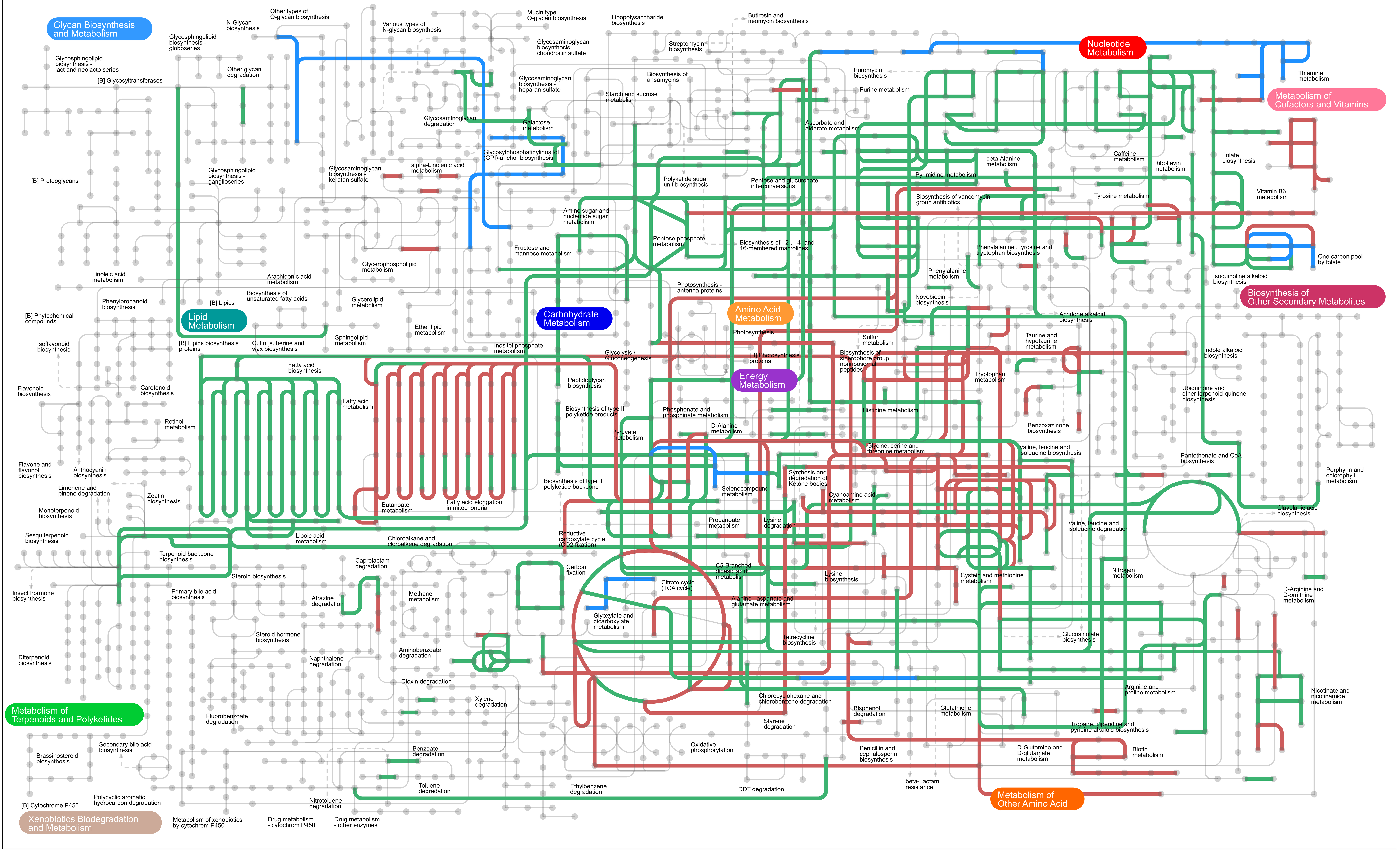

Glycan Biosynthesis and Metabolism
N-Glycan biosynthesis
Other types of O-glycan biosynthesis
Various types of N-glycan biosynthesis
Mucin type O-glycan biosynthesis
Lipopolysaccharide biosynthesis
Butirosin and neomycin biosynthesis
Nucleotide Metabolism
Glycosphingolipid biosynthesis - globoseries
Glycosaminoglycan biosynthesis - chondroitin sulfate
Streptomycin biosynthesis
Glycosphingolipid biosynthesis - lact and neolacto series
Other glycan degradation
Puromycin biosynthesis
Thiamine metabolism
[B] Glycosyltransferases
Glycosaminoglycan biosynthesis - heparan sulfate
Biosynthesis of ansamycins
Purine metabolism
Starch and sucrose metabolism
Metabolism of Cofactors and Vitamins
Glycosaminoglycan degradation
Galactose metabolism
Ascorbate and aldarate metabolism
Caffeine metabolism
Folate biosynthesis
Glycosylphosphatidylinositol (GPI)-anchor biosynthesis
beta-Alanine metabolism
Riboflavin metabolism
Glycosphingolipid biosynthesis - ganglioseries
Glycosaminoglycan biosynthesis - keratan sulfate
alpha-Linolenic acid metabolism
Pyrimidine metabolism
[B] Proteoglycans
Polyketide sugar unit biosynthesis
Pentose and glucuronate interconversions
Vitamin B6 metabolism
Biosynthesis of vancomycin group antibiotics
Tyrosine metabolism
Amino sugar and nucleotide sugar metabolism
Pentose phosphate metabolism
Fructose and mannose metabolism
Biosynthesis of 12-, 14- and 16-membered macrolides
Phenylalanine, tyrosine and tryptophan biosynthesis
One carbon pool by folate
Glycerophospholipid metabolism
Linoleic acid metabolism
Arachidonic acid metabolism
Phenylalanine metabolism
Isoquinoline alkaloid biosynthesis
Photosynthesis - antenna proteins
Biosynthesis of unsaturated fatty acids
Biosynthesis of Other Secondary Metabolites
Phenylpropanoid biosynthesis
[B] Lipids
Glycerolipid metabolism
Novobiocin biosynthesis
Lipid Metabolism
Carbohydrate Metabolism
Amino Acid Metabolism
[B] Phytochemical compounds
Acridone alkaloid biosynthesis
Ether lipid metabolism
Photosynthesis
Sphingolipid metabolism
Sulfur metabolism
Taurine and hypotaurine metabolism
Isoflavonoid biosynthesis
[B] Lipids biosynthesis proteins
Cutin, suberine and wax biosynthesis
Inositol phosphate metabolism
Indole alkaloid biosynthesis
Glycolysis / Gluconeogenesis
[B] Photosynthesis proteins
Biosynthesis of siderophore group nonribosomal peptides
Fatty acid biosynthesis
Tryptophan metabolism
Energy Metabolism
Peptidoglycan biosynthesis
Flavonoid biosynthesis
Carotenoid biosynthesis
Ubiquinone and other terpenoid-quinone biosynthesis
Fatty acid metabolism
Biosynthesis of type II polyketide products
Phosphonate and phosphinate metabolism
Histidine metabolism
Retinol metabolism
D-Alanine metabolism
Benzoxazinone biosynthesis
Pyruvate metabolism
Glycine, serine and threonine metabolism
Valine, leucine and isoleucine biosynthesis
Pantothenate and CoA biosynthesis
Flavone and flavonol biosynthesis
Anthocyanin biosynthesis
Synthesis and degradation of Ketone bodies
Porphyrin and chlorophyll metabolism
Biosynthesis of type II polyketide backbone
Limonene and pinene degradation
Selenocompound metabolism
Cyanoamino acid metabolism
Zeatin biosynthesis
Butanoate metabolism
Fatty acid elongation in mitochondria
Clavulanic acid biosynthesis
Monoterpenoid biosynthesis
Propanoate metabolism
Lysine degradation
Valine, leucine and isoleucine degradation
Sesquiterpenoid biosynthesis
Lipoic acid metabolism
Chloroalkane and chloroalkene degradation
Reductive carboxylate cycle (CO2 fixation)
Terpenoid backbone biosynthesis
Caprolactam degradation
C5-Branched dibasic acid metabolism
Carbon fixation
Nitrogen metabolism
Steroid biosynthesis
Citrate cycle (TCA cycle)
Lysine biosynthesis
Cystein and methionine metabolism
Insect hormone biosynthesis
Primary bile acid biosynthesis
Atrazine degradation
Methane metabolism
D-Arginine and D-ornithine metabolism
Alanine, aspartate and glutamate metabolism
Glyoxylate and dicarboxylate metabolism
Steroid hormone biosynthesis
Tetracycline biosynthesis
Glucosinolate biosynthesis
Aminobenzoate degradation
Diterpenoid biosynthesis
Naphthalene degradation
Dioxin degradation
Xylene degradation
Arginine and proline metabolism
Nicotinate and nicotinamide metabolism
Metabolism of Terpenoids and Polyketides
Chlorocyclohexane and chlorobenzene degradation
Bisphenol degradation
Glutathione metabolism
Fluorobenzoate degradation
Styrene degradation
Tropane, piperidine and pyridine alkaloid biosynthesis
Secondary bile acid biosynthesis
Brassinosteroid biosynthesis
Benzoate degradation
Oxidative phosphorylation
Penicillin and cephalosporin biosynthesis
D-Glutamine and D-glutamate metabolism
Biotin metabolism
Toluene degradation
Ethylbenzene degradation
beta-Lactam resistance
Polycyclic aromatic hydrocarbon degradation
DDT degradation
Metabolism of Other Amino Acid
[B] Cytochrome P450
Nitrotoluene degradation
Xenobiotics Biodegradation and Metabolism
Metabolism of xenobiotics by cytochrom P450
Drug metabolism - cytochrom P450
Drug metabolism - other enzymes

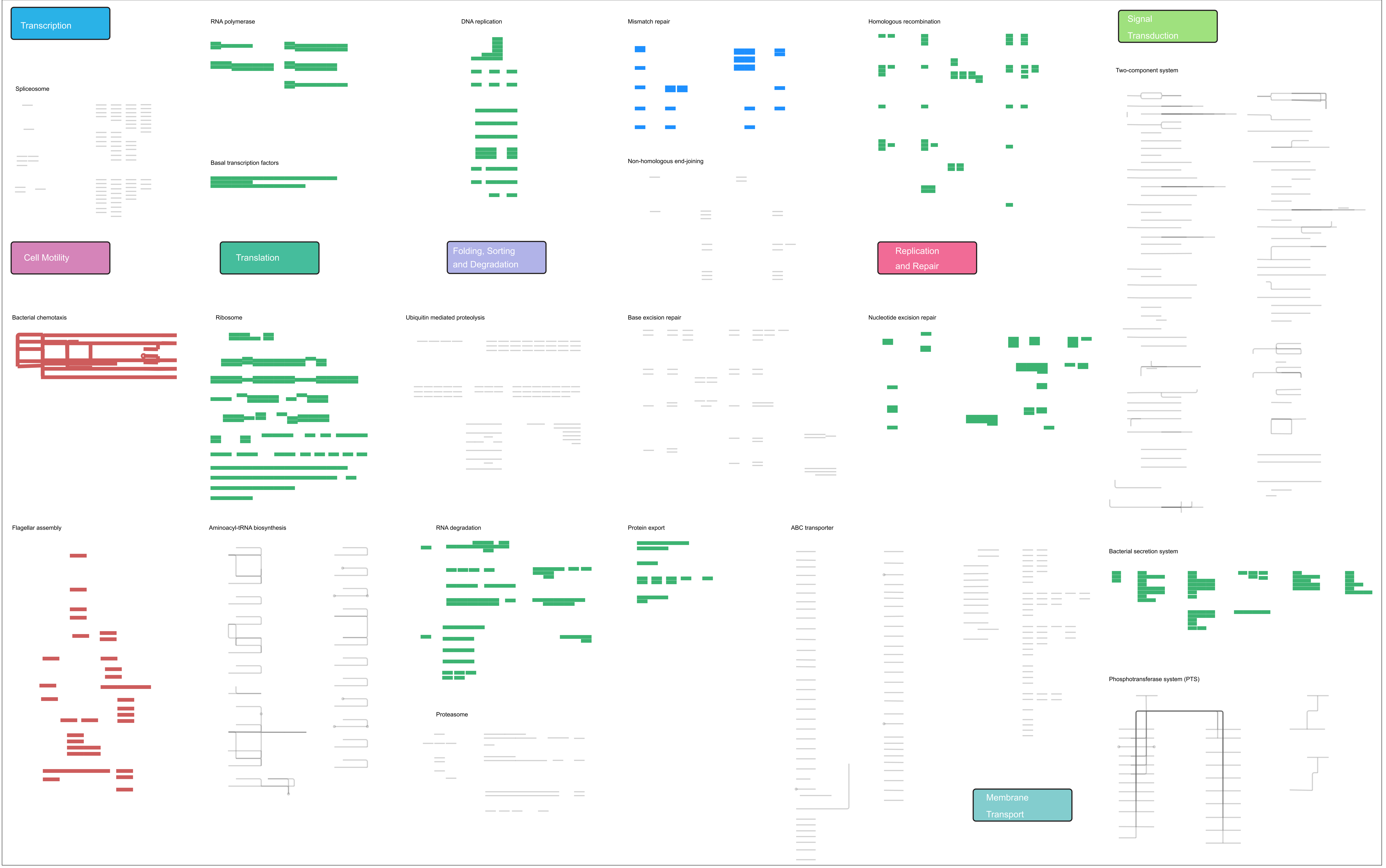
Transcription
RNA polymerase
DNA replication
Mismatch repair
Homologous recombination
Signal Transduction
Two-component system
Spliceosome
Basal transcription factors
Non-homologous end-joining
Cell Motility
Translation
Folding, Sorting and Degradation
Replication and Repair
Bacterial chemotaxis
Ribosome
Ubiquitin mediated proteolysis
Base excision repair
Nucleotide excision repair
Flagellar assembly
Aminoacyl-tRNA biosynthesis
RNA degradation
Protein export
ABC transporter
Bacterial secretion system
Proteasome
Phosphotransferase system (PTS)
Membrane Transport

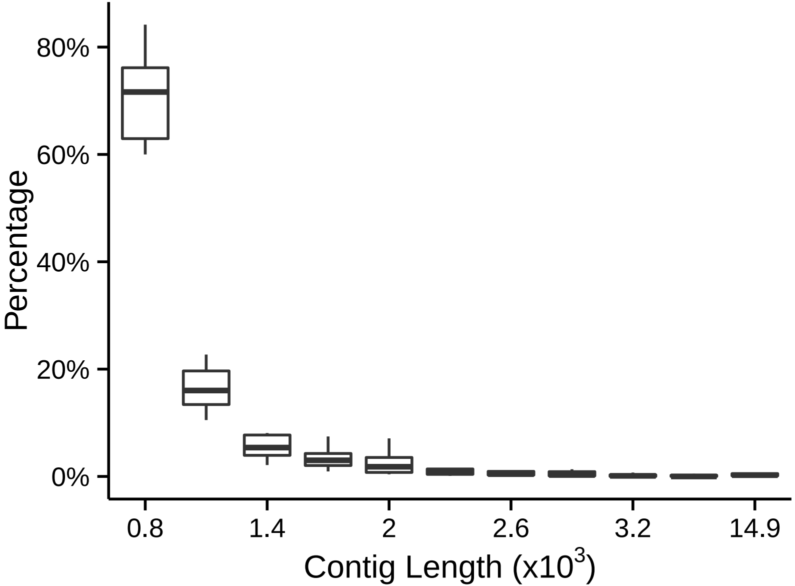
80%
60%
40%
20%
0%
Percentage
0.8
1.4
2
2.6
3.2
14.9
Contig Length (x10^3)

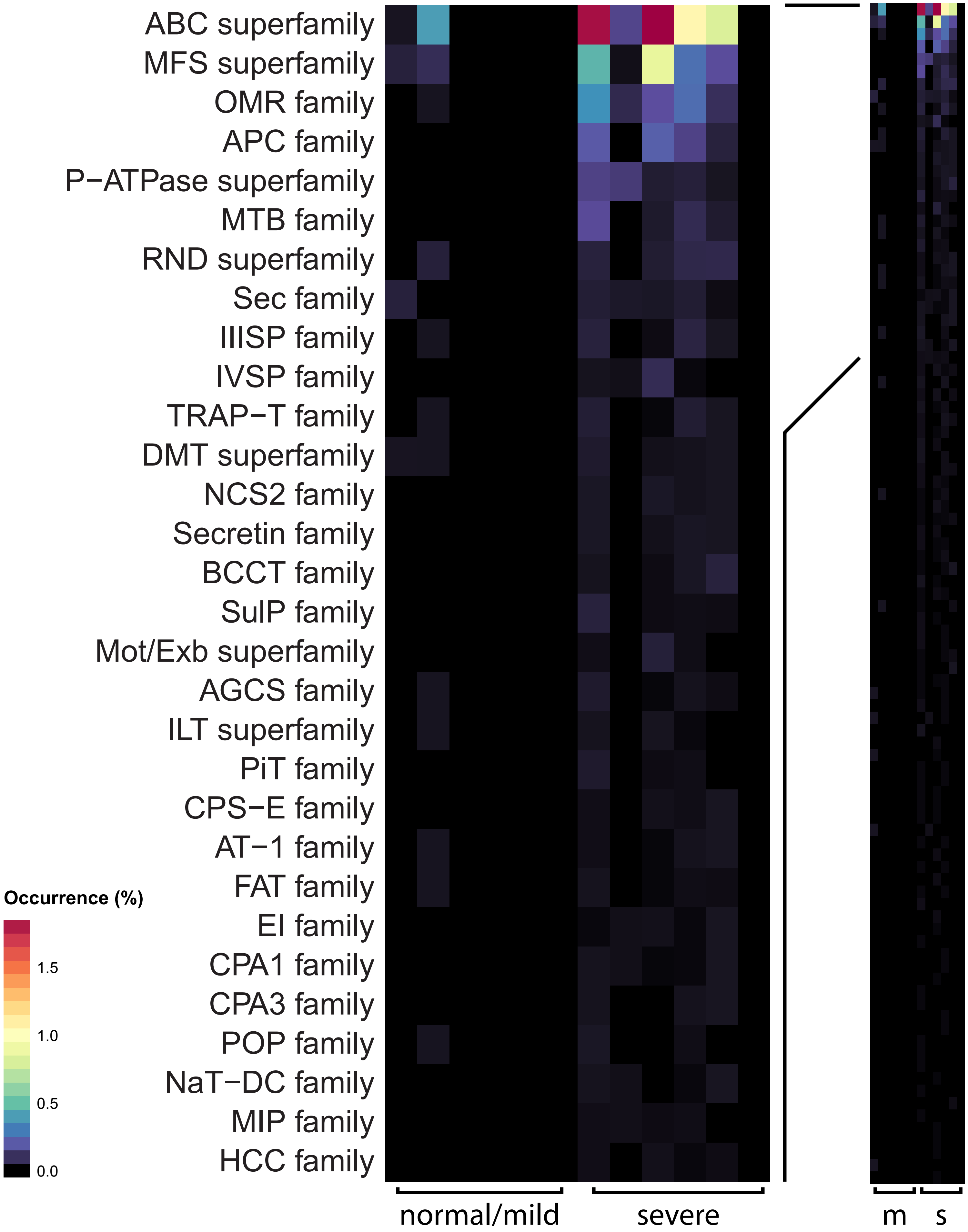
ABC superfamily
MFS superfamily
OMR family
APC family
P−ATPase superfamily
MTB family
RND superfamily
Sec family
IIISP family
IVSP family
TRAP−T family
DMT superfamily
NCS2 family
Secretin family
BCCT family
SulP family
Mot/Exb superfamily
AGCS family
ILT superfamily
PiT family
CPS−E family
AT−1 family
FAT family
EI family
CPA1 family
CPA3 family
POP family
NaT−DC family
MIP family
HCC family
Occurrence (%)
1.5
1.0
0.5
0.0
normal/mild
severe
m
s

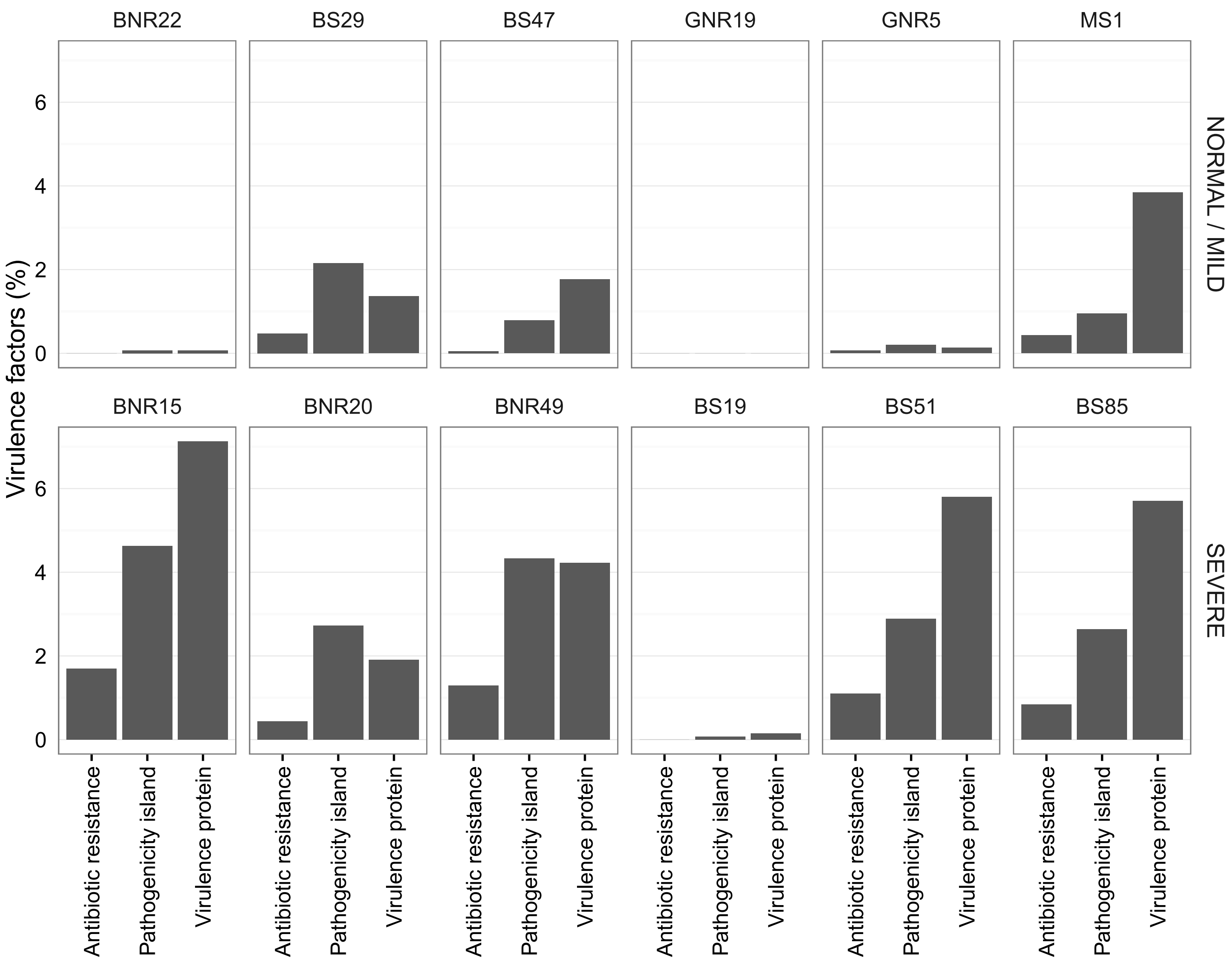

BNR22
BS29
BS47
GNR19
GNR5
MS1
BNR15
BNR20
BNR49
BS19
BS51
BS85
Virulence factors (%)
0
2
4
6
NORMAL / MILD
SEVERE
Antibiotic resistance
Pathogenicity island
Virulence protein

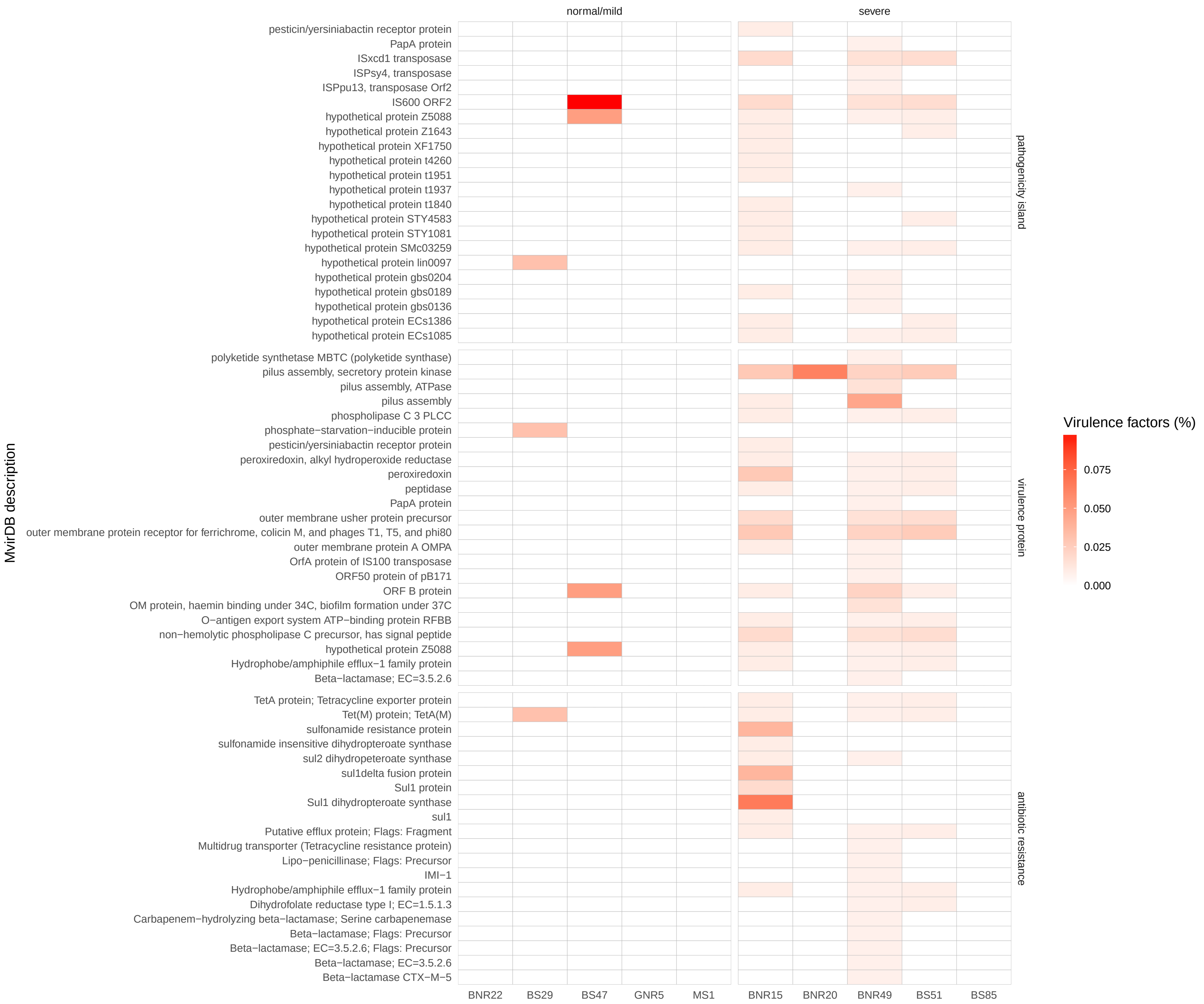
normal/mild
severe
pesticin/yersiniabactin receptor protein
PapA protein
ISxcd1 transposase
ISPsy4, transposase
ISPpu13, transposase Orf2
IS600 ORF2
hypothetical protein Z5088
hypothetical protein Z1643
hypothetical protein XF1750
hypothetical protein t4260
hypothetical protein t1951
hypothetical protein t1937
hypothetical protein t1840
hypothetical protein STY4583
hypothetical protein STY1081
hypothetical protein SMc03259
hypothetical protein lin0097
hypothetical protein gbs0204
hypothetical protein gbs0189
hypothetical protein gbs0136
hypothetical protein ECs1386
hypothetical protein ECs1085
polyketide synthetase MBTC (polyketide synthase)
pilus assembly, secretory protein kinase
pilus assembly, ATPase
pilus assembly
phospholipase C 3 PLCC
phosphate−starvation−inducible protein
pesticin/yersiniabactin receptor protein
peroxiredoxin, alkyl hydroperoxide reductase
peroxiredoxin
peptidase
PapA protein
outer membrane usher protein precursor
outer membrane protein receptor for ferrichrome, colicin M, and phages T1, T5, and phi80
outer membrane protein A OMPA
OrfA protein of IS100 transposase
ORF50 protein of pB171
ORF B protein
OM protein, haemin binding under 34C, biofilm formation under 37C
O−antigen export system ATP−binding protein RFBB
non−hemolytic phospholipase C precursor, has signal peptide
hypothetical protein Z5088
Hydrophobe/amphiphile efflux−1 family protein
Beta−lactamase; EC=3.5.2.6
TetA protein; Tetracycline exporter protein
Tet(M) protein; TetA(M)
sulfonamide resistance protein
sulfonamide insensitive dihydropteroate synthase
sul2 dihydropeteroate synthase
sul1delta fusion protein
Sul1 protein
Sul1 dihydropteroate synthase
sul1
Putative efflux protein; Flags: Fragment
Multidrug transporter (Tetracycline resistance protein)
Lipo−penicillinase; Flags: Precursor
IMI−1
Hydrophobe/amphiphile efflux−1 family protein
Dihydrofolate reductase type I; EC=1.5.1.3
Carbapenem−hydrolyzing beta−lactamase; Serine carbapenemase
Beta−lactamase; Flags: Precursor
Beta−lactamase; EC=3.5.2.6; Flags: Precursor
Beta−lactamase; EC=3.5.2.6
Beta−lactamase CTX−M−5
MvirDB description
BNR22
BS29
BS47
GNR5
MS1
BNR15
BNR20
BNR49
BS51
BS85
pathogenicity island
virulence protein
antibiotic resistance
Virulence factors (%)
0.075
0.050
0.025
0.000

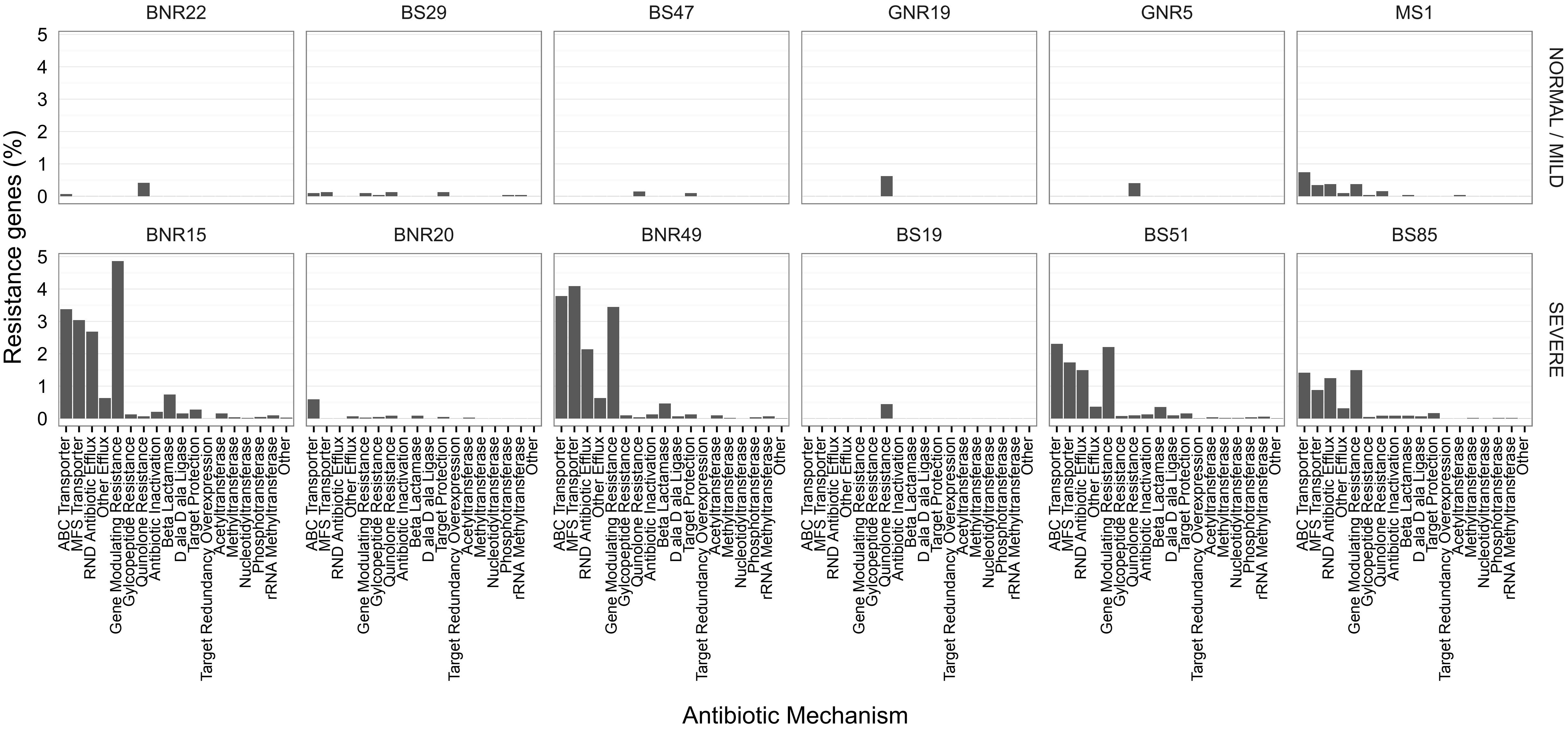

BNR22
BS29
BS47
GNR19
GNR5
MS1
BNR15
BNR20
BNR49
BS19
BS51
BS85
NORMAL / MILD
SEVERE
Resistance genes (%)
0
1
2
3
4
5
ABC Transporter
MFS Transporter
RND Antibiotic Efflux
Other Efflux
Gene Modulating Resistance
Gylcopeptide Resistance
Quinolone Resistance
Antibiotic Inactivation
Beta Lactamase
D ala D ala Ligase
Target Protection
Target Redundancy Overexpression
Acetyltransferase
Methyltransferase
Nucleotidyltransferase
Phosphotransferase
rRNA Methyltransferase
Other
Antibiotic Mechanism

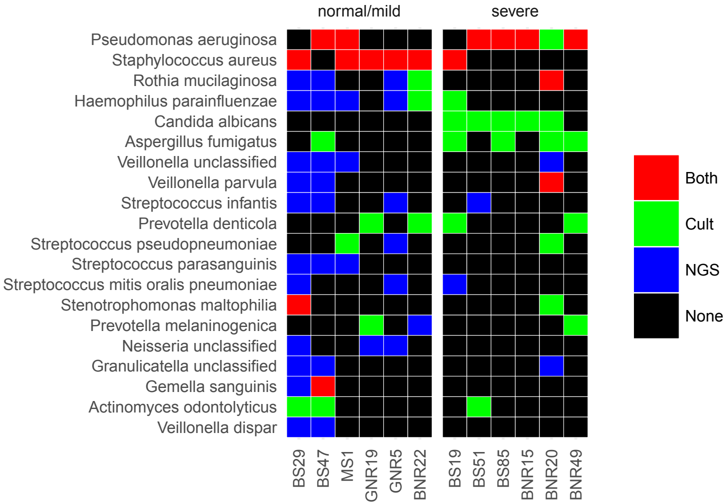

normal/mild
severe
Pseudomonas aeruginosa
Staphylococcus aureus
Rothia mucilaginosa
Haemophilus parainfluenzae
Candida albicans
Aspergillus fumigatus
Veillonella unclassified
Veillonella parvula
Streptococcus infantis
Prevotella denticola
Streptococcus pseudopneumoniae
Streptococcus parasanguinis
Streptococcus mitis oralis pneumoniae
Stenotrophomonas maltophilia
Prevotella melaninogenica
Neisseria unclassified
Granulicatella unclassified
Gemella sanguinis
Actinomyces odontolyticus
Veillonella dispar
BS29
BS47
MS1
GNR19
GNR5
BNR22
BS19
BS51
BS85
BNR15
BNR20
BNR49
Both
Cult
NGS
None

Table S1. Culture-based diagnostic microbiology of sputum from patients with CF.

Study ID	Lung disease status[1]	CF-related pathogens	*P. aeruginosa*/ MSSA/MRSA colonization[2]
BS29	normal/mild	*Stenotrophomonas maltophilia*, MSSA[3]	Intermittent colonization with *P. aeruginosa* (from 2010)
BS47	normal/mild	*Pseudomonas aeruginosa*	Chronic colonization with multi-drug resistance (MDR) *P. aeruginosa* (before 2000)
MS1	normal/mild	*P. aeruginosa* (mucoid phenotype), *Staphylococcus aureus*	Chronic colonization with *P. aeruginosa* and intermittent colonization with *S. aureus* (from 2011)
GNR19	normal/mild	MSSA, *Ralstonia* spp.	Intermittent colonization with MSSA (before 2005)
GNR5	normal/mild	MSSA, *Pseudomonas* spp.	Intermittent colonization with MSSA (before 2005) and *P. aeruginosa* (from 2007)
BNR22	normal/mild	MSSA	Chronic colonization with MSSA (before 2000)
BS19	severe	MRSA[4], MSSA	Chronic colonization with MRSA; (before 2000) Intermittent colonization with MSSA (from 2003)
BS51	severe	*P. aeruginosa* (mucoid phenotype), *Achromobacter xylosoxidans*	Chronic colonization with MDR *P. aeruginosa* (before 2000)
BS85	severe	*P. aeruginosa* (mucoid phenotype), *P. aeruginosa* (small phenotype)	Chronic colonization with MDR *P. aeruginosa* (before 2000)
BNR15	severe	MRSA, *P. aeruginosa* (mucoid phenotype), *P. aeruginosa* (frayed phenotype), *P. aeruginosa* (small colony), *P. aeruginosa* (round phenotype)	Chronic colonization with MDR *P. aeruginosa* (before 2000) and MRSA (before 2000)
BNR20	severe	*P. aeruginosa* (mucoid phenotype), *P. aeruginosa* (punctiform phenotype), *P. aeruginosa* (frayed phenotype), *S. maltophilia*	Chronic colonization with MDR *P. aeruginosa* (from 2011) and *S. maltophilia* (from 2003)
BNR49	severe	*P. aeruginosa* (mucoid phenotype), *P. aeruginosa* (frayed phenotype)	Chronic colonization with MDR *P. aeruginosa* (from 2010)

[1] Normal/mild, $FEV_1 > 70\%$; severe, $FEV_1 < 40\%$.
[2] In parentheses, the year of first acquisition of CF pathogen.
[3] MSSA, methicillin-susceptible *Staphylococcus aureus*
[4] MRSA, methicillin-resistant *Staphylococcus aureus*

Table S2. Number of metagenomic sequences obtained and processed for each patient.

Sample ID	Lung disease status[1]	# of reads	bacterial reads, %	# of bacterial reads	# of contigs	# of contigs, ≥ 500bp	filtered contigs, %	N50	coverage (avg. ± st. error)	# of ORF
BS29	normal/ mild	15005000	2.87	430546	19297	959	4.97	697	3.81 ± 0.63	3153
BS47	normal/ mild	15005000	2.89	433770	12778	420	3.29	714	14.01 ± 3.42	2035
MS1	normal/ mild	15005000	2.62	393657	18859	800	4.24	652	3.2 ± 0.36	3252
GNR19	normal/ mild	15005000	1.48	222641	4532	252	5.56	741	11.83 ± 2.71	1127
GNR5	normal/ mild	15005000	2.1	315463	8356	305	3.65	817	13.77 ± 2.71	1487
BNR22	normal/ mild	15005000	2.4	360642	8895	329	3.7	797	6.07 ± 0.92	1471
BS19	severe	15005000	3.36	503666	7440	271	3.64	908	11.91 ± 2.19	1352
BS51	severe	15005000	3.53	529435	23062	4223	18.31	806	2.45 ± 0.21	11326
BS85	severe	15005000	2.74	411701	14291	1922	13.45	686	2.48 ± 0.33	6170
BNR15	severe	15005000	2.87	430430	12245	4085	33.36	1655	2.46 ± 0.19	10710
BNR20	severe	15005000	4.55	682833	17545	995	5.67	645	4.04 ± 0.61	4774
BNR49	severe	15005000	4.87	731105	52370	5302	10.12	1199	2.91 ± 0.16	13318

Table S3. Mantel test P values and R statistics obtained comparing all biotic features. In the right part of the table, the values of the R statistics were reported whereas the P values were reported in the left part. T=taxonomic; K=KEGG pathways; E=gene frequencies (eggNOG database); V=virulence factors (MvirDB database); A=antibiotic resistance genes (Resfam database).

R statistic / P value	T	K	E	V	A
**T**		0.285	0.571	0.510	0.345
**K**	0.026		0.159	0.175	0.219
**E**	0.003	0.163		0.873	0.619
**V**	0.001	0.169	0.001		0.478
**A**	0.008	0.025	0.001	0.002	

Table S4. Variable loadings on the PCs across samples. The top-50% variables were reported in bold.

**Antibiotic Resistance**	**A1**	**A2**				
exp*	0.808	0.072				
ABC Transporter	0.248	-0.126				
Acetyltransferase	0.237	-0.220				
Antibiotic Inactivation	0.259	0.024				
Beta Lactamase	0.256	-0.049				
D ala D ala Ligase	0.254	0.109				
Gene Modulating Resistance	0.259	-0.107				
Gylcopeptide Resistance	0.254	-0.026				
Methyltransferase	0.252	0.025				
MFS Transporter	0.237	-0.210				
Nucleotidyltransferase	0.207	0.471				
Other	0.238	0.01				
Other Efflux	0.251	-0.149				
Phosphotransferase	0.234	0.131				
Quinolone Resistance	-0.163	0.075				
RND Antibiotic Efflux	0.256	-0.088				
rRNA Methyltransferase	0.254	0.024				
Target Protection	0.229	0.043				
Target Redundancy Overexpression	0.086	0.766				
Functional Categories	F1	F2	F3	F4	F5	F6
exp*	0.417	0.146	0.127	0.081	0.075	0.065
Translation ribosomal structure and biogenesis	-0.068	-0.078	0.512	-0.262	-0.138	-0.016
RNA processing and modification	-0.192	0.056	-0.032	-0.024	0.09	0.617
Transcription	-0.237	-0.186	-0.072	0.331	0.222	0.110
Replication recombination and repair	0.288	-0.095	0.172	0.145	-0.039	-0.110
Chromatin structure and dynamics	-0.161	0.166	-0.256	-0.263	0.062	-0.462
Cell cycle control cell division chromosome partitioning	-0.132	-0.222	0.313	0.099	-0.391	-0.145
Defense mechanisms	-0.193	-0.009	0.234	-0.228	0.129	0.200
Signal transduction mechanisms	-0.217	0.132	0.09	0.474	-0.136	0.165
Cell wall membrane envelope biogenesis	0.314	0.117	-0.053	-0.036	0.075	0.024
Cell motility	-0.297	0.007	0.051	0.159	-0.096	-0.123
Cytoskeleton	-0.09	-0.054	-0.075	0.146	0.527	-0.085
Extracellular structures	-0.063	-0.300	0.208	0.277	0.350	-0.327
Intracellular trafficking secretion and vesicular transport	-0.191	-0.372	-0.119	-0.198	0.019	-0.120
Posttranslational modification protein turnover chaperones	-0.310	0.021	0.003	-0.068	-0.144	-0.152
Energy production and conversion	-0.219	0.293	-0.265	-0.09	0.053	-0.124
Carbohydrate transport and metabolism	-0.029	-0.468	-0.276	-0.074	0.029	0.022
Amino acid transport and metabolism	-0.189	0.289	0.195	-0.307	0.230	0.068
Nucleotide transport and metabolism	0.038	-0.273	-0.433	-0.093	-0.250	0.219
Coenzyme transport and metabolism	-0.298	-0.101	0.027	-0.230	0.014	-0.063
Lipid transport and metabolism	-0.311	-0.045	0.026	0.062	0.147	0.163
Inorganic ion transport and metabolism	-0.273	-0.024	-0.097	0.047	-0.358	-0.014

Secondary metabolites biosynthesis transport and catabolism	-0.123	0.364	-0.159	0.318	-0.157	-0.164

Metabolic Pathways	M1	M2	M3	M4	M5
exp*	0.397	0.224	0.185	0.093	0.039
Glycosylphosphatidylinositol GPI anchor biosynthesis	0.194	-0.040	-0.140	0.008	-0.027
Alanine aspartate and glutamate metabolism	-0.181	0.122	0.144	-0.045	0.099
Synthesis and degradation of ketone bodies	-0.171	0.188	-0.052	0.068	0.061
Nicotinate and nicotinamide metabolism	-0.064	-0.113	0.239	0.015	-0.153
Other glycan degradation	-0.008	-0.257	0.153	0.128	0.022
Selenocompound metabolism	0.068	0.164	0.258	-0.056	-0.036
Glycine serine and threonine metabolism	-0.214	0.066	0.103	-0.005	0.004
Homologous recombination	0.064	-0.144	0.275	0.042	0.101
Vitamin B6 metabolism	-0.199	0.021	0.152	-0.029	-0.093
Fatty acid biosynthesis	-0.119	0.172	0.191	0.022	0.132
Bacterial secretion system	-0.173	0.177	0.084	-0.042	0.023
Pyruvate metabolism	0.192	0.156	0.073	-0.021	-0.056
Cysteine and methionine metabolism	-0.121	-0.207	-0.168	0.049	0.027
Riboflavin metabolism	0.111	0.003	0.197	0.121	-0.189
Protein export	-0.143	-0.186	-0.103	0.159	0.095
Nitrotoluene degradation	0.056	-0.089	-0.133	-0.350	-0.250
Thiamine metabolism	0.223	-0.025	0.002	-0.075	-0.017
RNA degradation	0.17	0.136	0.115	0.061	0.139
Geraniol degradation	-0.150	0.224	-0.068	-0.013	-0.012
Ribosome	-0.068	-0.188	0.043	-0.336	0.026
Carbon fixation pathways in prokaryotes	-0.200	0.064	0.146	0.021	-0.043
Valine leucine and isoleucine biosynthesis	-0.098	-0.122	0.092	-0.324	0.192
beta Alanine metabolism	-0.109	-0.116	-0.258	0.098	0.009
Folate biosynthesis	-0.123	-0.099	0.174	0.109	-0.198
Citrate cycle TCA cycle	0.147	0.188	0.101	-0.097	-0.166
Histidine metabolism	0.008	0.262	0.129	0.093	-0.157
Lipoic acid metabolism	-0.071	-0.156	-0.241	0.172	0.071
Peptidoglycan biosynthesis	0.045	-0.052	0.258	-0.134	0.342
Butanoate metabolism	-0.184	0.147	-0.062	-0.138	-0.01
DNA replication	0.217	0.053	0.051	-0.074	-0.121
Bacterial chemotaxis	-0.122	0.251	-0.034	-0.036	-0.132
Biotin metabolism	-0.209	0.033	0.042	0.05	-0.223
Pentose phosphate pathway	-0.008	-0.139	0.146	-0.335	-0.120
Sulfur relay system	-0.162	-0.127	0.025	0.009	-0.384
Basal transcription factors	0.168	-0.071	0.017	0.136	-0.340
C5 Branched dibasic acid metabolism	-0.106	-0.142	0.025	-0.344	-0.083
RNA polymerase	-0.153	-0.075	0.133	-0.150	-0.168
Mismatch repair	0.197	0.117	0.108	0.061	0.009
Terpenoid backbone biosynthesis	-0.125	-0.113	0.211	0.184	-0.07
Flagellar assembly	-0.167	0.193	-0.088	-0.034	0.019
D Glutamine and D glutamate metabolism	-0.093	-0.105	0.218	0.132	0.314
D Alanine metabolism	0.086	0.061	-0.06	-0.336	0.172
Valine leucine and isoleucine degradation	-0.172	0.201	-0.042	-0.032	-0.024

Pyrimidine metabolism	-0.095	-0.149	0.244	0.120	0.051
Nucleotide excision repair	0.206	0.119	0.07	0.044	-0.003
One carbon pool by folate	0.201	-0.005	0.135	-0.026	0.024
Caprolactam degradation	-0.096	0.240	-0.092	-0.031	0.096
Virulence Factors	V1				
exp*	0.852				
Differential gene regulation	-0.446				
Pathogenicity island	-0.416				
Protein toxin	-0.447				
Transcription factor	-0.448				
Virulence protein	-0.478				

* Proportion of variance explained
